# Prevention of Delayed Bleeding after Endoscopic Resection using Gel Hemostatic Powder: A Multicentric Open-lapel Prospective Study

**DOI:** 10.1055/a-2933-9485

**Published:** 2026-09-03

**Authors:** Julia C. Rodriguez, Pierre H. Deprez, Raf Bisschops, Pieter Dewint, Pierre Eisendrath, Christophe Snauwaert, Dominiek De Wulf, Philippe Leclercq, Jacques Deviere, Vincent Huberty, Ana Maria Bucalau, Mariana Figueiredo Ferreira, Sohaib Ouazzani, Marianna Arvanitaki, Arnaud Lemmers

**Affiliations:** 1 Department of Gastroenterology, Hepatopancreatology, and Digestive OncologyErasme Hospital, Hôpital Universitaire de Bruxelles (HUB), Université Libre de Bruxelles (ULB)BrusselsBrusselsBelgium; 2 Department of HepatogastroenterologyCliniques Universitaires Saint-Luc, Université Catholique de LouvainBruxellesBruxellesBelgium; 3 Department of Gastroenterology and HepatologyUniversity Hospital LeuvenLeuvenBelgium; 4 Department of Gastroenterology and Hepatology 82428 AZ Maria MiddelaresSint-NiklaasFlandersBelgium; 5 Department of Gastroenterology, Hepatopancreatology, and Digestive OncologyCHU Saint Pierre, Université Libre de BruxellesBrusselsBelgium; 6 Department of Hepatology and Gastroenterology 60208 Sint-Jan Brugge General HospitalBrugesFlandersBelgium; 7 Department of Gastroenterology and Hepatology 192827 AZ DeltaRoeselareFlandersBelgium; 8 Department of Gastroenterology and Hepatology 60182 University Hospitals LeuvenLeuvenFlandersBelgium

**Keywords:** endoscopy upper GI tract, endoscopic resection (ESD, EMR …), endoscopy lower GI tract, polyps/adenomas/…, non-variceal bleeding, precancerous conditions and cancerous lesions (displasia and cancer) stomach, lower GI bleeding

## Abstract

**Background** Delayed bleeding (DB) after endoscopic mucosal resection (EMR) and endoscopic submucosal dissection (ESD) remains a significant clinical problem. This study aimed to evaluate the safety and efficacy of a new gel hemostatic powder (GHP) for the prevention of DB in high-risk patients.

**Methods** A prospective, multicentric, open-label study was conducted at seven Belgian hospitals from 2023 to 2024 in patients undergoing EMR or ESD for lesions over 20 mm with high risk of DB (either anticoagulant or P2Y12RA treated, or undergoing duodenal EMR). The Nexpowder GHP preventive spray was applied to the resected area at the end of the procedure. The primary outcome was the rate of DB within 4 weeks post-procedure. Secondary outcomes included safety, procedure duration, post-procedural pain, and incidence of adverse events.

**Results** Fifty patients were enrolled in the study and treated with endoscopic resection followed by GHP application. The observed DB rate was 22% (11/50), higher than the literature-reported rate of 16%. The hypothesized reduction in DB below 5% was not achieved ( *p* = 0.2472). DB was most common in rectal ESD ( *n* = 3/5; 60%) and duodenal EMR ( *n* = 6/20; 30%). DB typically occurred within the first 10 days post-procedure (median onset 7d). On univariate analysis, the only factor associated with DB risk was lesion size ( *p* = 0.042). No adverse events related to the use of the GHP were reported.

**Conclusion** In this prospective study, GHP demonstrated limited efficacy for the prevention of DB in high-risk patients, with a 22% observed DB rate. Lesion size and location were key predictors of DB.

## Introduction

Endoscopic mucosal resection (EMR) and endoscopic submucosal dissection (ESD) are established procedures for the removal of neoplastic lesions in the gastrointestinal (GI) tract. These techniques are preferred over former traditional surgical methods of resection due to their organ preserving nature and a more favorable patient mortality and morbidity profile. However, one of the main adverse events (AEs) that does occur with endoscopic resection is delayed bleeding (DB), defined as bleeding occurring after the procedure. DB occurs in 1–5% of cases according to previously published studies. 1 2 3

A major risk factor for DB is antithrombotic therapy. Patients undergoing antithrombotic treatment (anticoagulant or antiplatelet therapy) have a higher risk of postoperative bleeding after EMR or ESD. Studies have shown that patients receiving this type of medication, particularly those taking direct oral anticoagulants (DOACs), experience higher DB rates after colorectal ESD, with one study reporting a rate of 17.2% in anticoagulant users and 23.3% in DOAC users compared to 11.4% in patients receiving warfarin. 3 Similarly, another study reported that the DB rate in patients receiving anticoagulants was 11.8%, compared to 6.6% in patients without antithrombotic therapy. 2

Regarding antiplatelet therapy, one study led by Okayama University Hospital reported that the use of P2Y12 receptor antagonists (P2Y12RAs) was a significant risk factor for DB after gastric ESD. 4 Additional independent risk factors for DB were lesion size greater than 12 mm and anticoagulant use. 4 In gastric EMR and ESD, a systematic review and meta-analysis confirmed that lesion size over 20 mm was a significant risk factor for DB. 5 Furthermore, Fanning et al. reported that duodenal EMR of larger lesions (>30 mm) was significantly associated with an increased risk of DB. 6

The location of the lesion may also influence the risk of DB. For example, duodenal EMR carries a substantial risk of AEs, including bleeding. A prospective study that enrolled 110 patients with duodenal adenomas reported a DB rate of 18.6%, and a significantly higher risk was associated with lesions ≥30 mm. 7

Efforts have been made to mitigate bleeding complications and endoscopic hemostatic techniques, such as prophylactic mucosal defect clipping, are widely used. However, even if recommended after right-side colon EMR, no technique has been proven to be cost-effective for the prevention of DB in high-risk patients. 8 9 10 A meta-analysis performed by Spadaccini et al. 11 supported the notion that prophylactic clipping should not be routinely recommended, but could be proposed for a subgroup of higher-risk lesions or patients. Rather than applying the procedure systematically, this approach would more effectively decrease both AEs and associated costs.

Nevertheless, innovative solutions to prevent DB have been developed recently. 12 Several studies have investigated the efficacy of gel hemostatic powders (GHPs), 17 18 19 including the Nexpowder™ endoscopic hemostasis system, a non-contact hemostatic spray designed to form a sticky gel upon contact with fluids, for the treatment of active bleeding. Although attractive in this indication, with its provision of a sticky coating that is formed on resected fields, no data are available on the use of GHP to prevent DB after endoscopic resection.

Considering its ease of use and its sticky behavior, the present prospective study aimed to assess the safety and efficacy of using GHP for the prevention of DB in high-risk patients undergoing EMR or ESD for large esophageal, gastric, duodenal, or colorectal lesions.

## Materials and Methods

### Study Design

This was an academic, multicentric, prospective, national, open-label, non-controlled interventional study designed to evaluate the safety and efficacy of a GHP (Nexpowder™ system, sold by Medtronic, Minneapolis, Minnesota, USA) for the prevention of DB in high-risk patients undergoing ESD or EMR for lesions over 20 mm. The study included patients from seven participating centers across Belgium (CUB Erasme Hospital, HUB [Brussels], UZ Leuven [Leuven], Cliniques Universitaires St-Luc [Brussels], AZ Maria-Middelares [Ghent], CHU St-Pierre [Brussels], AZ Sint-Jan [Brugge-Oostende], and AZ Delta [Roeselare]), with enrollment conducted from September 2023 to June 2024, to reach the statistically determined target of 50 patients.

### Inclusion Criteria

Eligible patients were over 18 years old with documented lesions measuring over 20 mm with an indication for endoscopic removal by ESD or EMR who were receiving anticoagulant (e.g., vitamin K antagonists, DOAC, unfractionated heparin) and/or antithrombotic P2Y12RA therapy. Also included were patients undergoing duodenal EMR without anticoagulation.

**Fig. 1:**
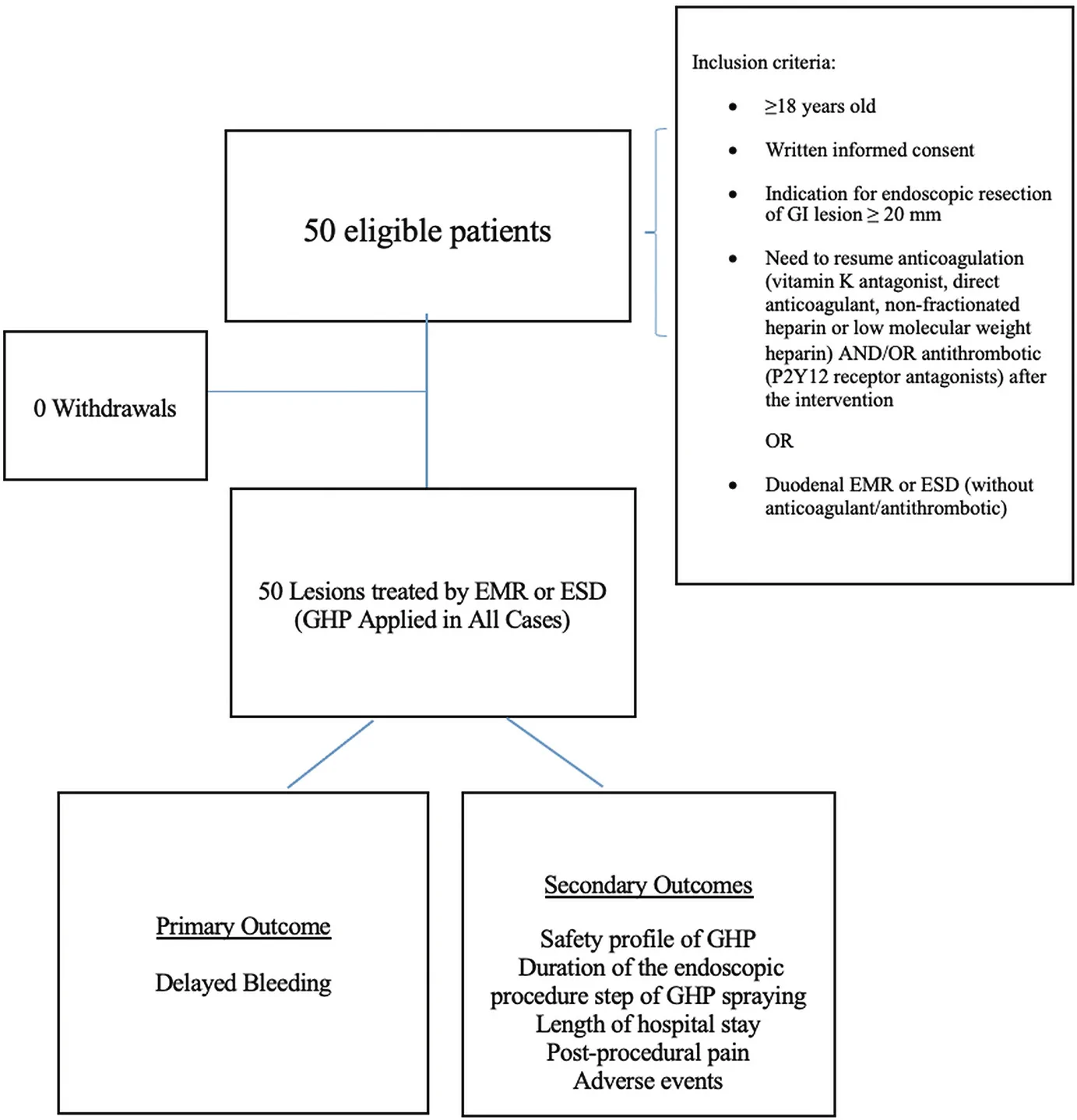
Study flowchart and design overview.

The flowchart of the study design is shown in Fig. 1 .

All patients provided written informed consent. The study protocol was approved by every participating center’s IRB and registered at Clinicaltrials.gov under the number NCT06096948.

### Sample Size Calculation

Based on data from the literature, the rate of DB after endoscopic resection in all organs in the absence of anticoagulants is about 1.2%, and in the presence of anticoagulant or P2Y12RA therapy is about 16%. In the duodenum, the DB rate has been reported to be higher than 5%, even in the absence of antithrombotic therapy. 15 16 17 18 19

Using a one-sided Fisher’s exact test, the sample size was calculated to detect a reduction in the DB rate from a literature-reported rate of 16–3.7%, with a power of 80% and an α of 0.05 (Supplementary Data; Tables 1 and 2 ).

**Table 1:** Clinical and endoscopic characteristics of patients with or without delayed bleeding.

Variable	No delayed bleeding ( *N* = 39)	Delayed bleeding ( *N* = 11)	*p* -Value
Gender			>0.9
Male	29 (74%)	8 (73%)	
Female	10 (26%)	3 (27%)	
Age (y)	73 (68–79)	75 (58–82)	0.8
Anti-P2Y12			0.6
No	34 (89%)	11 (100%)	
Yes	4 (11%)	0 (0%)	
Anticoagulant (DOAC, heparin, vitamin K antagonists)			0.3
No	14 (36%)	6 (55%)	
Yes	25 (64%)	5 (45%)	
Coagulation tests			
Platelet count (/mm ^3^ )	210000 (157000–241000)	147000 (5435–231500)	0.3
INR	1.01 (0.97–1.05)	1.03 (0.98–1.09)	0.5
APTT (s)	27.30 (24.60–29.30)	31.00 (25.10–31.00)	0.3
Type of resection			0.5
ESD	19 (49%)	4 (36%)	
Conventional EMR	20 (51%)	7 (64%)	
Cold snare EMR	0	0	
Location of lesion			0.073
Colon	9 (23%)	1 (9.1%)	
Duodenum	14 (36%)	6 (55%)	
Esophagus	9 (23%)	0 (0%)	
Rectum	2 (5.1%)	3 (27%)	
Stomach	5 (13%)	1 (9.1%)	
Size of the lesion			
Lesion length (mm) (median [range])	30 (20–40)	40 (30–65)	0.037
Lesion width (mm)	25 (20–33)	40 (20–40)	0.2
En-Bloc resection			0.6
No	18 (46%)	6 (55%)	
Yes	21 (54%)	5 (45%)	
Endoscopic complete resection			>0.9
No	1 (2.6%)	0 (0%)	
Yes	38 (97%)	11 (100%)	
Intraprocedural complications			>0.9
No	36 (92%)	10 (91%)	
Yes	3 (7.7%)	1 (9.1%)	
Intraprocedural bleeding			0.7
No	15 (38%)	3 (27%)	
Yes	24 (62%)	8 (73%)	
Type of bleeding			0.3
Oozing	20 (83%)	5 (63%)	
Severe non-pulsating	3 (13%)	2 (25%)	
Severe pulsating	1 (4.2%)	1 (13%)	
Hemostasis during procedure			0.7
No	15 (38%)	4 (36%)	
Yes	24 (62%)	7 (64%)	
Type of hemostasis During procedure			
Use of coagulation forceps			>0.9
No	32 (82%)	9 (82%)	
Yes	7 (18%)	2 (18%)	
Coagulation with ESD knife			0.7
No	28 (72%)	9 (82%)	
Yes	11 (28%)	2 (18%)	
Coagulation with closed tip of snare			0.6
No	35 (90%)	9 (82%)	
Yes	4 (10%)	2 (18%)	
Use of clips for hemostasis			0.5
No	37 (95%)	10 (91%)	
Yes	2 (5.1%)	1 (9.1%)	
Methods to prevent delayed bleeding			
Preventive vessels coagulation			0.7
No	19 (49%)	6 (55%)	
Yes	20 (51%)	5 (45%)	
Closure of mucosal defect			0.4
No	28 (74%)	10 (91%)	
Yes	10 (26%)	1 (9.1%)	
Estimation of success of closure			>0.9
100%	3 (30%)	0 (0%)	
75–100%	4 (40%)	1 (100%)	
50–75%	1 (10%)	0 (0%)	
<50%	2 (20%)	0 (0%)	
GHP application			0.3
Estimation of coverage			
100%	32 (84%)	8 (73%)	
80–100%	5 (13%)	2 (18%)	
40–60%	1 (2.6%)	0 (0%)	
Number of grams used	3.00 (3.00–3.00)	3.00 (3.00–3.00)	0.6
Procedure duration			>0.9
Total procedure duration (min)	75 (43–107)	80 (62–107)	
Duration of GHP spraying (sec)	50 (35–73)	68 (59–143)	0.10

*N* (%); median (IQR), quantitative variables are described using medians and interquartile ranges (IQRs).

Statistics were computed using Fisher’s exact test, Wilcoxon rank sum test.

APTT, activated partial thromboplastic time; DOAC, direct oral anticoagulant; EMR, endoscopic mucosal resection; ESD, endoscopic submucosal dissection; GHP, gel hemostatic powder; INR, international normalized ratio.

**Table 2:** Delayed bleeding rates by resection method and anatomical location.

Type of resection and location	Number of patients ( *N* = 50)	Delayed bleeding ( *N* = 11)
ESD	**23 (46%)**	**17% (4/23)**
Esophagus	9 (18%)	0% (0/9)
Stomach	5 (10%)	20% (1/5)
Duodenum	1 (2%)	0% (0/1)
Rectum	5 (10%)	60% (3/5)
Colon	3 (6%)	0% (0/3)
Conventional EMR	**27 (54%)**	**26% (7/27)**
Stomach	1 (14%)	0% (0/1)
Duodenum	19 (38%)	30% (6/19)
Colon	7 (14%)	14% (1/7)

EMR, endoscopic mucosal resection; ESD, endoscopic submucosal dissection.

### Treatment and Procedures

The endoscopic resection procedure was performed under general anesthesia with orotracheal intubation or under sedation following indication and center policy. The technical modalities of the resection (ESD or EMR) were performed according to the endoscopist’s preference.

We restricted inclusion to lesions >20 mm, as lesions of this size are typically managed with advanced endoscopic resection techniques (EMR or ESD) in routine clinical practice.

**Fig. 2:**
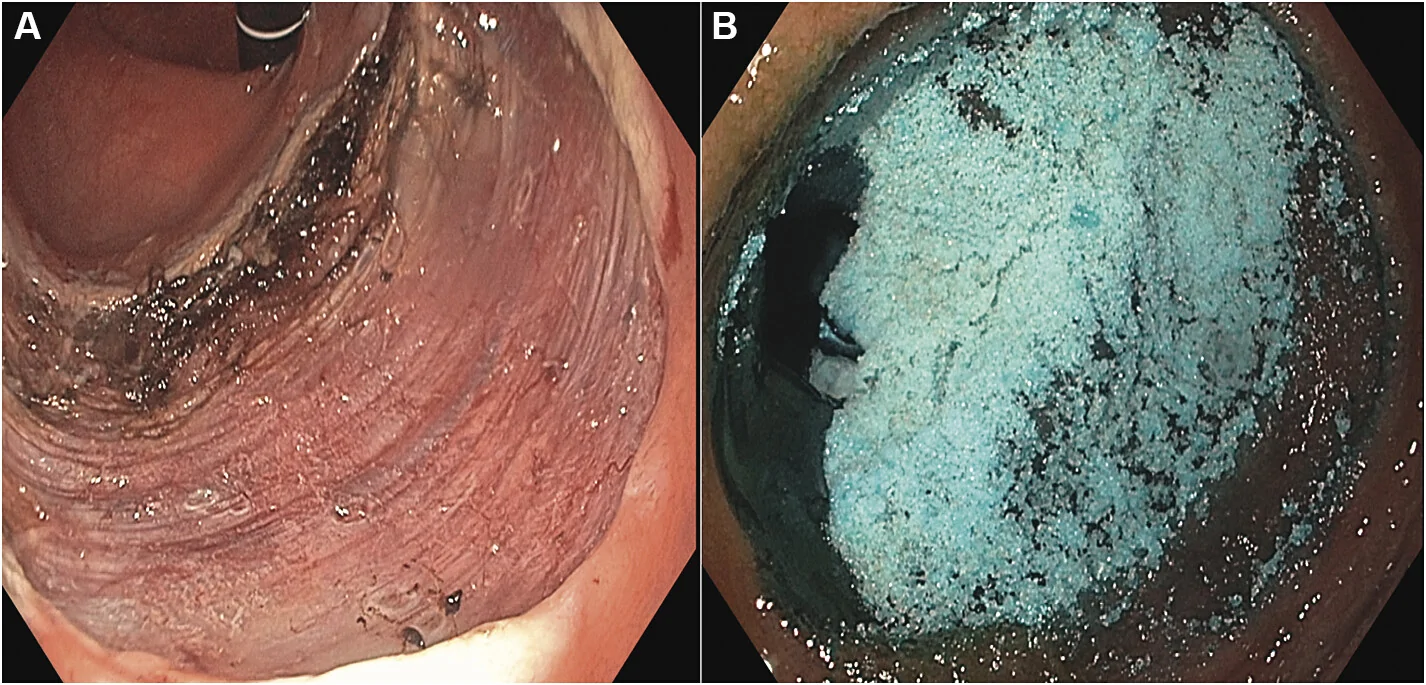
( **A** ) Mucosal defect after resection. ( **B** ) GHP sprayed on the mucosal defect zone. GHP, gel hemostatic powder.

Regarding the center’s and the operator experience, all procedures in this study were performed by experienced, certified endoscopists who each has performed more than 100 advanced endoscopic resection procedures. Each center involved in the study is accredited for performing advanced procedures such as EMR and ESD. Several resection techniques were used by the endoscopists according to lesion characteristics and operator preference, including different EMR approaches (conventional and underwater EMR), hot-snare pEMR, en-bloc hot EMR, as well as ESD with adjunctive techniques such as immersion or traction when appropriate. For peri-procedural bleeding, usual thermal or mechanical hemostatic measures were applied. At the end of the resection, as a prophylactic method, the GHP (Nexpowder™ system,) was sprayed with an attempt to cover the entire resected field ( Fig. 2 ). Additional methods to prevent DB, such as closing the defect with clips or coagulation of vessels were allowed, according to the endoscopist’s judgment and local policy. P2Y12RAs were suspended at 7 days prior to the endoscopic procedure, until 48 h after the procedure. Similarly, anticoagulants were discontinued at least 48 h before the procedure and resumed within 48 h after the procedure, at the discretion of the endoscopist. Heparine was discontinued 24 h prior to the procedure. Details of the resumption time of each antithrombotic time are available in **Supplementary Table 3** .

Patients remained hospitalized overnight and, on Day 1, clinical and biological follow-up assessments were conducted. In some patients, a routine follow-up endoscopy was performed based on the usual practices of the participating center.

### Outcome Measures

The primary outcome was the rate of DB occurring within the 4 weeks following the procedure. DB was defined as clinical evidence of bleeding such as hematochezia or hematemesis or hemoglobin level decrease of more than 2 g/dL within 28 days after EMR or ESD. 3 The hypothesis was that the use of the GHP would reduce the rate of DB from 16% (reported rate in the literature) to less than 5% (expected observed rate during the study). 3

Secondary outcomes included the safety profile of the use of the GHP, the duration of the endoscopic procedure and additional time necessary for GHP spraying, the length of hospital stay, post-procedural pain, and the incidence of AEs. AEs were categorized as peri-procedural, early (up to 24 h post-procedure), and late (up to 4 weeks post-procedure) and were categorized according to the CTCAE classification system (Common Terminology Criteria for AEs).

### Follow-up and Data Collection

Data on procedure-related variables, such as lesion size and location, method of resection, and the amount and coverage of the GHP were collected during the procedure.

Patients were followed up to Week 4, either through an outpatient visit or a phone call, to assess AEs, quality of life, and any clinical outcomes. Any post-procedural AEs, including DB, were documented, along with severity and the interventions required.

### Statistical Methods

Quantitative variables are described using medians and interquartile ranges (IQRs), while qualitative variables are represented by frequencies. A proportion test (one-sample *z* -test) was applied to compare the observed proportions of DB in the sample with theoretical proportions. The Mann–Whitney–Wilcoxon test was used to compare the medians of quantitative variables between patients who experienced bleeding and those who did not. For qualitative variables, comparisons between bleeding and non-bleeding patients were made using the Chi-square test and Fisher’s exact test. The probability of DB over time was analyzed using the Kaplan–Meier analysis. Univariate Cox regression was performed to assess the association between various factors (e.g., gender, age, antithrombotic therapy [antiaggregant and anticoagulants], lesion maximum and minimum size, lesion location, technique of resection, intraprocedural bleeding, peri-procedural hemostasis, preventive vessel coagulation, mucosal defect closure, total procedure duration, intraprocedural complications, pathological analysis), and the occurrence of DB. Hazard ratios (HRs) are reported with their 95% confidence intervals (CIs), and the *p* -value from the Wald test is provided. A significance level of 0.05 (two-sided) was used for all statistical analyses, which were conducted using RStudio (2023.12.1+402).

## Results

### Demographics, Lesion Characteristics, and Procedures

During the study period, 50 patients were enrolled, with a median age of 73 years (IQR: 66–79). The majority of the participants were male (74%). Most of the participants were receiving antithrombotic therapy, including 8% (4/50) on anti-P2Y12 therapy, and 60% (30/50) on anticoagulant therapy, primarily Apixaban.

Lesions were mainly located in the duodenum (40%), followed by the colon (20%), esophagus (18%), stomach (12%), and rectum (10%). The median length of the lesions was 30 (IQR: 20–40) mm, and the median width was 30 (IQR: 20–40) mm.

In total, 46% of the lesions were resected with ESD and 54% with conventional EMR. Sixty percent of the patients resumed anticoagulant therapy and 8% resumed anti-P2Y12 therapy after the ER procedure, with a median of 5 days for anticoagulants (DOAC) and 7 days for anti-P2Y12 therapy. All patients completed the follow-up period.

### Primary Outcome Analysis

Of the 50 patients, 11 (22%) experienced DB with the highest incidence occurring in the rectum ( *n* = 3/5 [60%]) and the duodenum ( *n* = 6/20; 30%). No DB was observed after esophageal resection. Table 1 summarizes the comparative analysis of clinical and endoscopic characteristics between patients with and without DB.

**Fig. 3:**
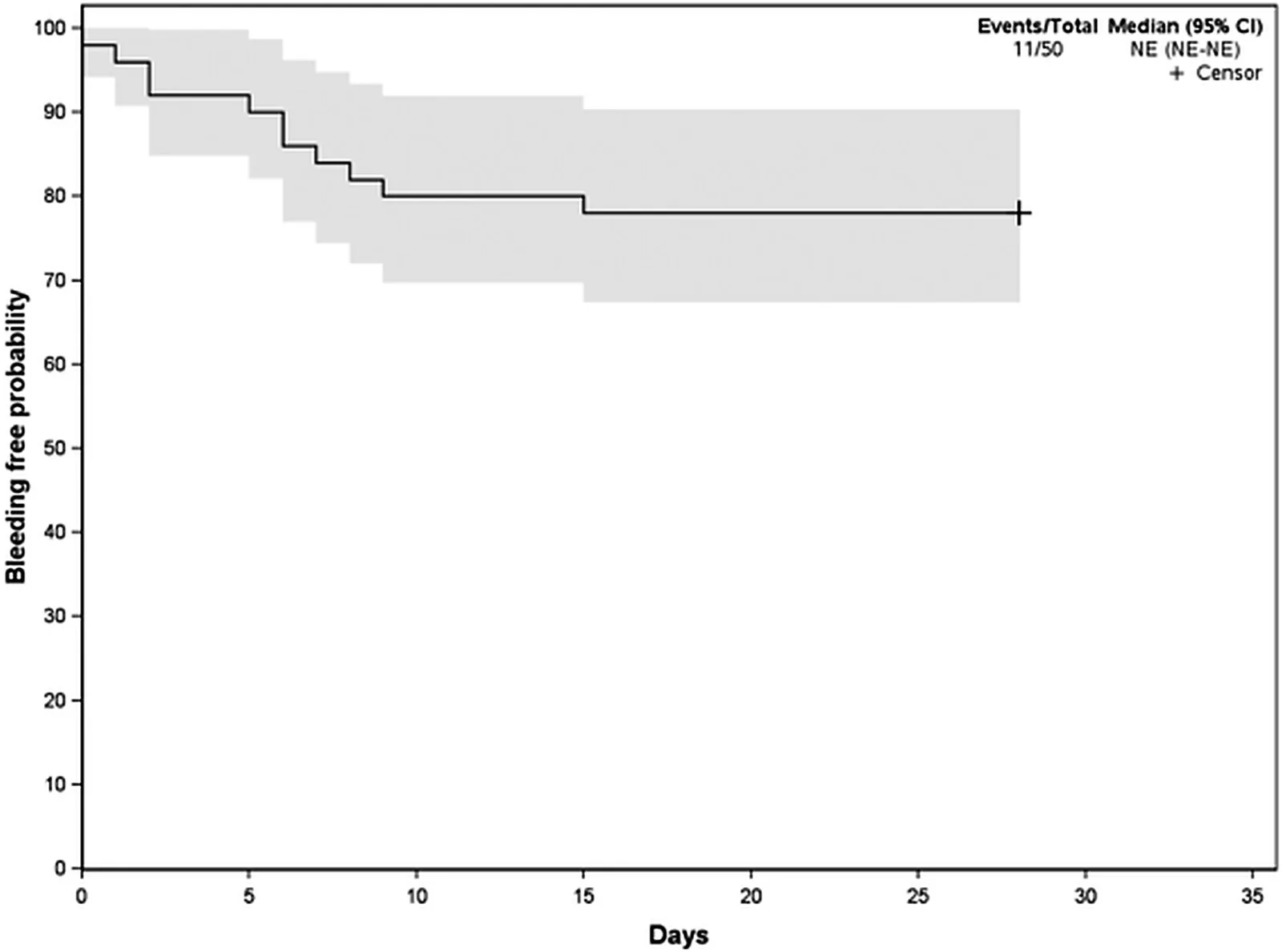
Bleeding Probability Survival Curve. This Kaplan–Meier curve illustrates the probability of bleeding over time (in days) among the 50 patients included in the study. Based on the Kaplan–Meier curve, the risk of bleeding appears to be most significant during the first 10 days after the procedure. This is evident from the sharpest decline in the curve, indicating that most of the bleeding events occurred early in the follow-up period. After day 10, the curve begins to plateau, suggesting that the risk of bleeding decreases and stabilizes over time, with fewer new bleeding events occurring as the day’s progress.

The Kaplan–Meier analysis indicated that the cumulative probability of bleeding risk increased particularly during the first 10 days post-procedure, as this was the case for 91% of the patients who presented with DB ( Fig. 3 ). Of the 11 patients who experienced DB, 5 (45%) were on anticoagulant treatment. Among these patients, two experienced bleeding before resuming their anticoagulant therapy and three had bleeding after resuming their treatment (48 h after procedure). The median time to onset of bleeding was 7 days.

The primary hypothesis (i.e., that the use of GHP would reduce the rate of DB from 16%, the reported rate in the literature, 3 to less than 5%) was not confirmed by the data from this cohort, as the observed DB rate using the GHP in this high-risk population (22% (95% CI: 12.8–35.2)) was higher than reported in the literature, although this difference was not statistically significant ( *p* = 0.2472).

Regarding the risk of bleeding according to the location of the lesion, patients treated with the GHP had a DB rate of 26.67% (95% CI: 10.9–51.95) in the colorectum, a DB rate of 16.67% (95% CI 3.01–56.35) in the stomach ( *n* = 6), and a DB rate of 30% (95% CI 14.55–51.99) in the duodenum ( *n* = 20) ( Table 2 ). These rates were compared to the reported literature in high-risk patients, without GHP treatment, as summarized in Table 3 .

**Table 3:** Rates of delayed bleeding by location: Comparison of the present GHP study results to those from the literature.

Delayed bleeding depending on location	Present GHP study	Literature (without GHP use)		
**Duodenum**	Present study	A. Probst 7	Fanning et al. 6	Binmoeller et al. 22
Delayed bleeding	30% (6/20)	18.6%	26.3%	25%
	*N* = 20	*N* = 118 EMR (<10–30 mm)	*N* = 19 EMR (>30 mm)	*N* = 12 EMR
**Colorectal**	Present study	Harada et al. 2	Ogiyama et al. 3	Yoshida et al. 20
Delayed bleeding	26.7% (4/15)	11.8% (anticoagulant group)	17.2%	11.7% (warfarin) 9.3% (DOAC)
	*N* = 15	*N* = 412 ESD	*N* = 87 ESD (100% anticoagulants)	*N* = 1478 ESD (100% anticoagulants)
**Stomach**	Present study	Nagami et al. 22	Hirai et al. 4	Kono et al. 1
Delayed bleeding	16.7% (1/6)	10.7% (antithrombotics)	20.6%	18%
	*N* = 6	*N* = 1353 ESD (100% antithrombotics)	*N* = 34 ESD (100% P2Y12RA)	*N* = 28 ESD (100% antithrombotics)

DOAC, direct oral anticoagulant; EMR, endoscopic mucosal resection; ESD, endoscopic submucosal dissection; GHP, gel hemostatic powder.

### DB Risk Factor Analysis

Univariate regression analysis was performed to identify factors associated with DB ( **Supplementary Table 4** ). The analysis showed that gender was not significantly associated with the risk of DB. The characteristics of the lesion, including size and location, were evaluated as potential predictors of DB. Specifically, lesion size was significantly associated with an increased risk of bleeding ( *p* = 0.042), with each additional millimeter in length increasing the risk by 2%. Lesion location also showed a trend toward risk, with rectal lesions presenting the highest HR compared to other locations, although this association did not reach statistical significance ( *p* = 0.07).

The use of daily P2Y12RA therapy and anticoagulant therapy did not demonstrate a significant impact on DB. The technique of resection (EMR vs. ESD) performed did not significantly impact DB rate. The total duration of the procedure was 75 min for non-bleeding patients and 80 min for those who experienced bleeding. Likewise, intraprocedural bleeding ( *n* = 32/50) and the need for hemostasis during the procedure were not significantly associated with DB. Preventive vessel coagulation at the end of the procedure ( *n* = 25/50) and attempts to close the mucosal defect ( *n* = 11/50) also did not significantly influence DB outcomes. Lesion type (e.g., benign, mucosal carcinoma, or submucosal carcinoma) was not significantly associated with DB.

Concerning the application of the GHP, a full 100% coverage across the resected field was achieved in 84% of cases with no DB and 73% of those who experienced DB ( *p* = 0.3).

### Secondary Outcomes Analysis

#### Pain

Pain, as assessed by general and abdominal pain scores, was low across all patients, with median scores of 0.0 in both patients presenting or not with DB, indicating minimal discomfort post-procedure ( *p* = 0.5).

#### Length of Stay

The median length of hospital stay was 1 day (IQR: 1.2). There was a larger IQR for the bleeding group (1–3 days) but this difference was not statistically significant ( *p* = 0.3). Out of the 11 patients who experienced DB, 5 (45%) were hospitalized with an average hospital stay of 2–4 days.

#### Duration of GHP Application

The duration of GHP application was longer in the bleeding group (median 68 s) compared to the non-bleeding group (median 50 s), though this difference did not reach statistical significance ( *p* = 0.10). This difference was likely attributable to the size of the mucosal defect.

#### Adverse Events

The analysis of early and late AEs revealed no significant association between these events and DB. During the procedure, perforations occurred in 4% of the cases and were managed endoscopically. Early and late AEs were experienced in 8% of the patients (e.g., dysphagia, mild acute pancreatitis, cardiac decompensation, and post-ESD stricture), and were related to the endoscopic procedure or the anesthesia. No patients were reported to have Grade 5 AEs (i.e., death related to an AE). No AEs related to the GHP application were observed.

## Discussion

This study aimed to assess the incidence of DB following the preventive use of a GHP in patients at high risk of bleeding after ESD or EMR. The findings were compared to the existing literature on DB in similar patient populations. This study using preventive GHP sprayed on resection fields observed a DB rate of 22% (11/50), which, although higher than the theoretical rate of 16% reported in patients on anticoagulant therapy, 3 did not significantly differ from it, suggesting that the application of the GHP is not helpful in preventing post-endoscopic resection DB in high-risk patients. Hypothetically, the higher DB rate observed in this study may be attributed to the inclusion of patients with larger lesions and those undergoing procedures in anatomically challenging locations such as the duodenum and rectum, where the risk of bleeding is the highest. 4 7 We specifically included lesions >20 mm and high-risk locations such as the duodenum to assess the efficacy of the GHP in a population with a known higher risk of DB. If we had included smaller lesions, the ones removed by cold snare polypectomy that are associated to less risk of DB, or lesions from lower-risk locations, the bleeding risk would not have been sufficient to evaluate the GHP effectiveness on a limited cohort.

Review of the literature stratified by specific location suggests that, without the use of preventive bleeding measures, DB rates are similar to those observed in this study, even if we aimed to prevent DB by spraying the GHP on the resection fields ( Table 3 ). A DB rate of 26.6% (4/15) was observed for colorectal lesions in this study. This rate is within the range of DB rates reported in the literature for similar procedures without GHP use. For example, a retrospective study on patients treated with anticoagulants undergoing colorectal ESD reported a DB rate of 9–12%. 20 In the duodenum, a similar scenario was observed with a DB rate of 30% (6/20) associated with the use of GHP, comparable to the 18.6–26.3% reported rates from the literature, without GHP use. 6 7 21 22 For gastric resection in patient treated with antithrombotics, the 16.7% (1/6) DB rate observed using the GHP in this study was similar to DB rates reported from the literature (10–20%) without the use of GHP. 1 4 22

A possible explanation for the lack of efficacy observed may be related to the duration of mucosal adherence of the GHP, which is estimated to last only 48–72 h. This timeframe is likely insufficient in the context of anticoagulant resumption and the timing of DB events, the majority of which occur within 10 days post procedure, often after day 3. As a result, the potential hemostatic effect of the GHP may no longer be active when the bleeding risk is higher.

Furthermore, the risk of DB in this study was significantly associated with the length of the lesion, with every 1 mm increase in lesion size correlating with a 2% increase in bleeding risk. This correlation is consistent with the existing literature, which identifies lesion size as a critical determinant of DB risk. Larger lesions, due to their extensive resection areas and the potential for more significant tissue damage, are inherently at higher risk for complications such as bleeding. 5 23 24 The importance of lesion size in predicting DB risk suggests the need for adapted interventions that take into account lesion characteristics and incorporate additional preventive measures for larger lesions. 1 25

The anatomical location of the lesion also played a role in DB rates in this study. In the rectum and the duodenum, the bleeding rates were the highest, at 60% (3/5) and 30% (6/20), respectively, and higher than rates reported in the literature. 7 23 26 In the case of the duodenum, this is likely related to its thin wall and rich vascularization and, as referred to earlier, can lead to increased DB rates despite the use of preventive measures such as clipping or vessel coagulation. 7 18

We acknowledge that duodenal lesions are known to have a higher risk of bleeding. The selection of such lesions in the inclusion criteria, even outside of the need for chronic treatment with antithrombotic for this indication was intentional to assess the efficacy of the GHP in a high-risk cohort, where the clinical need for improving patient care is crucial. The fact that for this location only, patients without antithrombotic treatment have been enrolled has probably introduced a shift to favor duodenal resection indications at the time of patient recruitment.

Numerous studies have shown that the use of anticoagulant and antiplatelet therapies contribute significantly to DB risk. 2 3 6 24 27 This study observed a higher, though not statistically significant, rate of DB among patients on anticoagulant therapy, which is consistent with published data. 2 3 6 The pharmacokinetics of DOACs, including their plasma concentration and Xa factor activity, have been closely linked to bleeding outcomes. 27 This pharmacological insight highlights the need for careful management of anticoagulation in high-risk endoscopic procedures.

No AEs were associated with the use of the GHP, confirming a favorable safety profile. However, perforations related to the procedure were observed in 4% of patients, consistent with the literature for ESD and EMR, particularly in high-risk anatomical locations. 4 7 18

In this study, the DB rate was 22% (11/50) with the use of GHP in high-risk patients, which demonstrates the limited effectiveness of this approach. Other endoscopic techniques, such as endoscopic hand suturing (EHS, SutuArt, Olympus), have demonstrated much more promising results. As reported in a prospective single-arm trial, 28 EHS led to a 0% DB rate in high-risk patients who underwent gastric ESD. The Overstitch endoscopic suturing device has also demonstrated efficacy in closing large defects post-ESD, preventing DB in a small retrospective study including low-risk patients in the absence of anticoagulant or antithrombotic therapy. 29 Another promising technique is the novel through-the-scope suturing system (X-Tack, Boston Scientific), which has demonstrated a high rate of complete closure and no DB events following the prophylactic closure of non-ampullary duodenal EMR defects. 30 Further studies are needed to confirm these data.

Regarding other topical hemostatic agents, the use of a self-assembling peptide (PuraStat™, 3D-Matrix) during ESD significantly reduced the need for thermal therapy in a randomized controlled trial, without having significant impact on the DB rate. 31 Also, although they demonstrate effective immediate hemostasis, hemostatic powders, including TC-325 (Hemospray, Cook, Bloomington, USA) and EndoClot (EndoClot Plus Santa Clara, California, USA), are still associated with significant rebleeding rates and variable impact on DB, limiting their long-term effectiveness. 12 13 14 The DB rates for various hemostatic powders range from 3.7% to 19.3%, depending on the location and severity of the initial bleeding. 14 32 However, the lack of prospective studies assessing the role of these hemostatic powders in the prevention of post-operative bleeding makes it difficult to compare those to GHP (Nexpowder™ system).

Finally, the limited efficacy of GHP in this study contrasts with the benefits of prophylactic clipping for preventing DB, mostly in high-risk patients with lesions over 20 mm. According to a meta-analysis conducted by Jiang et al., 33 prophylactic clipping demonstrates a statistically significant reduction in delayed DB, particularly for duodenal lesions larger than 20 mm (odds ratio: 0.35, 95% CI: 0.25–0.49, *P* < 0.01). Prophylactic clipping might be a more effective strategy for minimizing DB in similar patient populations. In this line, new anchor-type teeth clips (Mantis, Boston Scientific), designed to approximate resection margins, have been demonstrated to be associated with excellent closure capabilities in a short closure time (7–8 min) for defects up to 60 mm. 34 35

While GHP may have potential, its performance in this study suggests that it might need to be combined with other strategies, such as prophylactic clipping or thermal coagulation, to achieve optimal outcomes. This type of combined approach has been shown to significantly reduce DB rates, particularly in challenging anatomical sites like the duodenum and rectum. 33

There are some limitations in the current study. Mixing different endoscopic resection modalities (EMR and ESD) and lesion locations might lead to a heterogeneous population making subgroup analysis challenging and restricting generalization of the results. Furthermore, although, anticoagulant interruption and resumption were based on ESGE guidelines with specific recommendations for each type of anticoagulant, early follow-up protocols varied between centers. 10 Additional limitations include the variability of application of hemostasis preventive measures such as preventive clipping and closure of the defect at the discretion of the endoscopist, which could represent a bias. The sample size calculation was based on an assumed reduction in DB from 16% to 3.7%, which may have led to an overly optimistic expectation of the treatment effect. We recognize that this expected effect may have been too large, and may have reduced our ability to detect smaller yet clinically significant reductions in bleeding risk. On the other hand, despite the use of prophylactic vessel coagulation at the end of the resection in 50% (25/50) and clip closure of the mucosal defect in 22% (11/50), adding the GHP in this cohort was not associated with a low rate of DB. The DB rate might have been even higher if other preventive measures had not also been applied. The possibility to generalize these findings is thus limited, highlighting the need for further research. Future studies should focus on new gel hemostatic agents that stick to the resected field longer than 10 days to improve our toolbox for the prevention of DB in high-risk patients. 10 24 Finally, the lack of a contemporaneous control group limits the interpretability of the study. A more targeted randomized controlled trial focusing on specific locations could be a valuable next step.

In conclusion, this study demonstrated the challenges associated with managing DB in high-risk patients undergoing ESD or EMR, with a critical 10-day post-endoscopic resection period.

The single application of GHP in this high-risk cohort did not demonstrate a protective effect against DB. Further randomized studies or combined preventive strategies are needed to better assess its potential. These findings emphasize the need for personalized interventions based on lesion characteristics and patient risk factors, as well as additional research to optimize DB preventive strategies after endoscopic resection.

**Abbreviations**
AE: Adverse event
CI: Confidence interval
DB: Delayed bleeding
DOAC: Direct oral anticoagulant (or direct anticoagulant)
EHS: Endoscopic hand suturing
EMR: Endoscopic mucosal resection
ESD: Endoscopic submucosal dissection
GHP: Gel hemostatic powder
GI: Gastrointestinal
HR: Hazard ratio
IQR: Interquartile range
NCT: National Clinical Trial (refers to Clinicaltrials.gov number)
P2Y12RA: P2Y12 receptor antagonist

## Supplementary Material

Ergänzendes Material Supplementary Material
