# Supplementary material for: Prevention of Delayed Bleeding after Endoscopic Resection using Gel Hemostatic Powder: A Multicentric Open-lapel Prospective Study
Source: Endosc Int Open. 2026 Sep 3;14:a29339485. doi: 10.1055/a-2933-9485 (PMC13551450; doi:10.1055/a-2933-9485)
Supplement: Supplementary file 1 [file 10-1055-a-2933-9485_29376860.pdf]

Supplementary material

Julia Chaves Rodriguez[1], Pierre. H. Deprez[2], Raf Bisschops[3], Pieter Dewint[4], Pierre Eisendrath[1,5], Christophe Snauwaert[6], Dominiek De Wulf[7], Philippe Leclercq[3], Jacques Devière[1], Vincent Huberty[1], Ana Maria Bucalau[1], Mariana Figueiredo Ferreira[1,5], Sohaib Ouazzani[1], Marianna Arvanitakis[1], Arnaud Lemmers[1]

Prevention of delayed bleeding after endoscopic resection using gel hemostatic powder: a multicentric open-lapel prospective study

Power analysis

Delayed bleeding (DB) statistics from various organs (colon, rectum, stomach, and duodenum) were collected from the literature (Ref 1-10, 15–19) and are summarized in supplementary table 1.

Supplementary Table 1. Delayed bleeding rates according to anticoagulation status from the littérature review

Group	Delayed bleeding	Total lesions	Percentage
Without anticoagulation	62	5019	1.2
With anticoagulation	51	317	16.1
Total	113	5336	2.1

We designed a preliminary study to assess the benefit of the usage of GHP (Nexpowder™ system) in decreasing the percentage of DB. A number of 50 patients in the GHP group will be included in the study. Fixing a power of 80% and α= 0.05 (one-sided Fisher's Exact test), we will be able to assess up to 3.70% of DB in the GHP group (-77% of diminution between reference and GHP group) in the « with anticoagulation » group (Table 2). « Total » and « without anticoagulation » group didn't reach the statistical threshold as the percentages were already low (Figure1).

Supplementary Table 2. Power analysis for the anticoagulation cohort

Results for N=50 patients and power = 80%, α= 0.05 (one-sided Fisher's Exact test)

Group	Reference group percentage (cfr Table 1)	max GHP percentage to reach 80%	Percentage of DB diminution between GHP group and reference group
With anticoagulation	16.088	3.70	- 77%

Supplementary Table 3. Details of the mean time to resumption of each antithrombotic therapy by drug class

Antithrombotic therapy class	N	Mean time to treatment resumption (days)
NOACs (apixaban, rivaroxaban, edoxaban)	24	8.8
Vitamin K antagonist (Sintrom)	1	15.0
Heparins (Clexane, Fraxodi)	2	4.0
P2Y12 receptor antagonists (Clopidogrel)	2	10.5

Supplementary Table 4. Univariate regression analysis to identify factors associated with delayed bleeding

Characteristic	HR	95% CI	p-value
**Demographics**			
Gender			0.86
Male	—	—	
Female	1.13	0.30, 4.26	
Age	0.97	0.93, 1.01	0.16
**Antithrombotic Therapy**			0.15
Is the patient taking daily anti-P2Y12 (clopidogrel, prasugrel, ticagrelor)?			
No	—	—	
Yes	0.00	0.00, Inf	
**Anticoagulant Therapy**			0.19
Is the patient taking daily anticoagulant therapy (vitamin K antagonist, direct anticoagulant, non-fractionated heparin or low molecular weight heparin)?			
No	—	—	
Yes	0.45	0.14, 1.49	
**Lesion Characteristics**			
Size of the lesion (length in mm)	1.0	1.00, 1.04	0.042

	2		
Size of the lesion (width in mm)	1.03	0.98, 1.08	0.23
**Location of the Lesion**			0.070
Colon	—	—	
Duodenum	3.64	0.44, 30.2	
Esophagus	0.00	0.00, Inf	
Rectum	6.26	0.65, 60.2	
Stomach	1.65	0.10, 26.4	
**Type of Resection**			0.36
ESD	—	—	
Conventional EMR	1.75	0.51, 6.00	
Cold Snare EMR			
**Intraprocedural Bleeding**			0.51
No	—	—	
Yes	1.54	0.41, 5.82	
**Hemostasis during Resection Procedure**			0.68
No	—	—	
Yes	1.50	0.19, 11.7	
**Preventive Vessel Coagulation**			0.64
No	—	—	
Yes	0.76	0.23, 2.48	
**Mucosal Defect Closure**			0.22
No	—	—	
Yes	0.3	0.04, 2.58	

	3		
**Total Procedure Duration (minutes)**	1.00	0.99, 1.01	0.81
**Intraprocedural Complications**			0.83
No	—	—	
Yes	1.26	0.16, 9.85	
**Pathological Analysis**			0.20
Benign lesion	—	—	
Mucosal carcinoma	0.22	0.03, 1.74	
Submucosal carcinoma	0.48	0.06, 3.82	

Event N= Delayed bleeding
CI, confidence interval; EMR, endoscopic mucosal resection; ESD, endoscopic submucosal dissection; HR, hazard ratio,
